# Hippocampus long‐axis specialization throughout development: A meta‐analysis

**DOI:** 10.1002/hbm.26340

**Published:** 2023-05-20

**Authors:** Emily S. Nichols, Anna Blumenthal, Elizabeth Kuenzel, Jasmyn K. Skinner, Emma G. Duerden

**Affiliations:** ^1^ Faculty of Education Western University London Canada; ^2^ Western Institute for Neuroscience Western University London Canada; ^3^ Cervo Brain Research Centre Université Laval Quebec Canada; ^4^ Pediatrics, Schulich School of Medicine & Dentistry Western University London Canada

**Keywords:** development, fMRI, hippocampus, long‐axis specialization, meta‐analysis

## Abstract

The human adult hippocampus can be subdivided into the head, or anterior hippocampus and its body and tail, or posterior hippocampus, and a wealth of functional differences along the longitudinal axis have been reported. One line of literature emphasizes specialization for different aspects of cognition, whereas another emphasizes the unique role of the anterior hippocampus in emotional processing. While some research suggests that functional differences in memory between the anterior and posterior hippocampus appear early in development, it remains unclear whether this is also the case for functional differences in emotion processing. The goal of this meta‐analysis was to determine whether the long‐axis functional specialization observed in adults is present earlier in development. Using a quantitative meta‐analysis, long‐axis functional specialization was assessed using the data from 26 functional magnetic resonance imaging studies, which included 39 contrasts and 804 participants ranging in age from 4 to 21 years. Results indicated that emotion was more strongly localized to the anterior hippocampus, with memory being more strongly localized to the posterior hippocampus, demonstrating long‐axis specialization with regard to memory and emotion in children similar to that seen in adults. An additional analysis of laterality indicated that while memory was left dominant, emotion was processed bilaterally.

## Graphical abstract

The goal of this meta‐analysis was to determine whether the long‐axis functional specialization observed in adults is present earlier in development. Using a quantitative meta‐analysis, long‐axis functional specialization was assessed using the data from 26 functional magnetic resonance imaging studies, which included 39 contrasts and 804 participants ranging in age from 4 to 21 years. Results indicated that overall emotion was localized to the anterior hippocampus, with memory being localized to the posterior hippocampus, demonstrating long‐axis specialization with regard to memory and emotion in children similar to that seen in adults.

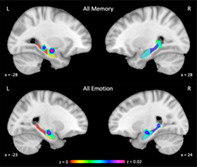

## 1 INTRODUCTION

The human adult hippocampus is differentially organized along its longitudinal axis. It can be subdivided into the head, or anterior hippocampus (aHPC) and its body and tail, or posterior hippocampus (pHPC). These regions have different connectivity patterns with cortical and subcortical brain regions and contain different compositions of the hippocampal subfields, with a higher proportion of CA1‐4 in the aHPC and a higher proportion of dentate gyrus in the pHPC. In addition, in rodents aHPC cells have larger receptive fields than those in the pHPC, which is thought to lead to differences in the gradient of representations, (i.e., more fine‐grained in the posterior [see Poppenk et al., 2013, for a review]). A recent functional magnetic resonance imaging (fMRI) study in humans reported converging evidence, with more coarse spatial and temporal representations in aHPC relative to pHPC (Brunec et al., 2018). In addition to major structural distinctions, a wealth of functional differences along the longitudinal axis have been reported. One line of literature emphasizes specialization for different aspects of cognition, whereas another emphasizes the unique role of the aHPC in emotional processing. Findings from a recent meta‐analysis of adult functional neuroimaging studies support both of these distinctions (Grady, 2020). However, although this functional organization is well‐established in adults and is becoming better understood, the timing of its emergence during childhood remains unknown.

Most existing research on structure and function of the hippocampus during development has been sparked by the paradox of childhood amnesia. Childhood amnesia refers to the finding that adults can recall very few episodic memories, that is, memories of life events, between the ages of 2 and 6 (Rubin, 2000), and is characterized by rapid forgetting of early memories (Bauer, 2015; Bauer & Larkina, 2014; Morris et al., 2010). This is a paradox, as children within this age range show many remarkable feats of memory despite having few memories of the actual events. Studies have demonstrated age‐related changes in episodic memory ability (Bauer et al., 2017; Bauer & Larkina, 2014; Newcombe et al., 2014), and this is thought to result from the protracted maturation of the hippocampus relative to neural systems that support other memory abilities (Lavenex & Banta Lavenex, 2013; Seress & Ábrahám, 2008).

Interestingly, in nonhuman primates, the rate of hippocampal maturation is not uniform across the longitudinal axis, with pHPC maturing later than aHPC (Lavenex & Banta Lavenex, 2013). In rodents, the ventral hippocampus (corresponding to the aHPC in humans) is mature by the second year of life (Jabès & Nelson, 2015). In contrast, the dorsal hippocampus (corresponding to the pHPC) begins to develop in the second year of life, and continues to develop until age five (Ghetti & Bunge, 2012; Gómez & Edgin, 2016). The slow maturation of the pHPC is thought to underlie childhood amnesia; that is, detailed episodic memory emerges alongside the protracted development of the pHPC (Hayne & Imuta, 2011; Scarf et al., 2013). In contrast, other types of learning, such as statistical learning and generalized memory are purported to rely on the earlier‐maturing aHPC, which also emerges earlier in development (Schlichting et al., 2017).

Thus, it appears that functional differences in cognition between the anterior and posterior HPC appear early in development, in‐line with HPC maturation, but it remains unclear whether this is also the case for functional differences in emotion processing. The central role of the aHPC in stress and affect has been emphasized in the nonhuman animal literature (see Fanselow & Dong, 2010, for a review) and the HPC is implicated in affective disorders (Malchow et al., 2015; Sheline et al., 2002). In adults, it is engaged during emotion regulation, motivation tasks, and processing of emotional faces (Barch et al., 2019; Kirby & Robinson, 2017; Pessoa, 2017). Ranganath and Ritchey (2012) propose the aHPC is part of an anterior‐medial system dedicated to processing people and things, with positive or negative affect being of core importance. A recent meta‐analysis supports this model, showing that in human adults, social, emotional, and face processing largely occur in the aHPC (Grady, 2020).

Similar to the functional specificity of the aHPC and pHPC in a development, little is known about lateralization of these processes within the hippocampus. Although historically emotion was proposed to be right‐lateralized, a recent meta‐analysis found evidence for right, left, and bilateral network activation in adults during emotion regulation, depending on task demands (Morawetz et al., 2020). Similarly, while studies directly examining lateralization of memory processing in the hippocampus are few, there is evidence in adults for both left and right hippocampal involvement depending on the task being performed (Coleshill et al., 2004; Maguire et al., 1997; J. Miller et al., 2018; Saling et al., 1993). In children, Hopf et al. (2013) found evidence for right lateralization of relational memory processing; however, the degree of left and right hippocampal involvement in other tasks in children remains unknown.

The functional organization of the aHPC and pHPC has not been explored in the developing brain. Thus, the goal of this meta‐analysis is to amalgamate existing developmental fMRI findings to determine whether the long‐axis functional specialization observed in adults is present earlier in development. Specifically, our objective is to localize cognitive functions within the hippocampus in typically developing children, and ask whether emotion functions are localized to the aHPC, mirroring that seen in adults. In addition, we explore whether functions are lateralized, and whether that differs across emotional and non‐emotional function. Ideally, we would also characterize the rich range of cognitive functions that have shown differential organization, such as that explored by Grady (2020). However, given the relatively smaller numbers of developmental neuroimaging studies with healthy children this was not possible at this time. Thus, we focused on emotion‐related activation and contrasted that with studies involving non‐emotional aspects of memory, using cognitive topics as defined by Poldrack et al. (2012). The outcome variable for this objective will be the average coordinates of each cognitive function listed above. The age range is birth to 21 years of age.

## 2 METHODS

### 2.1 Article selection and literature search

**FIGURE 1:**
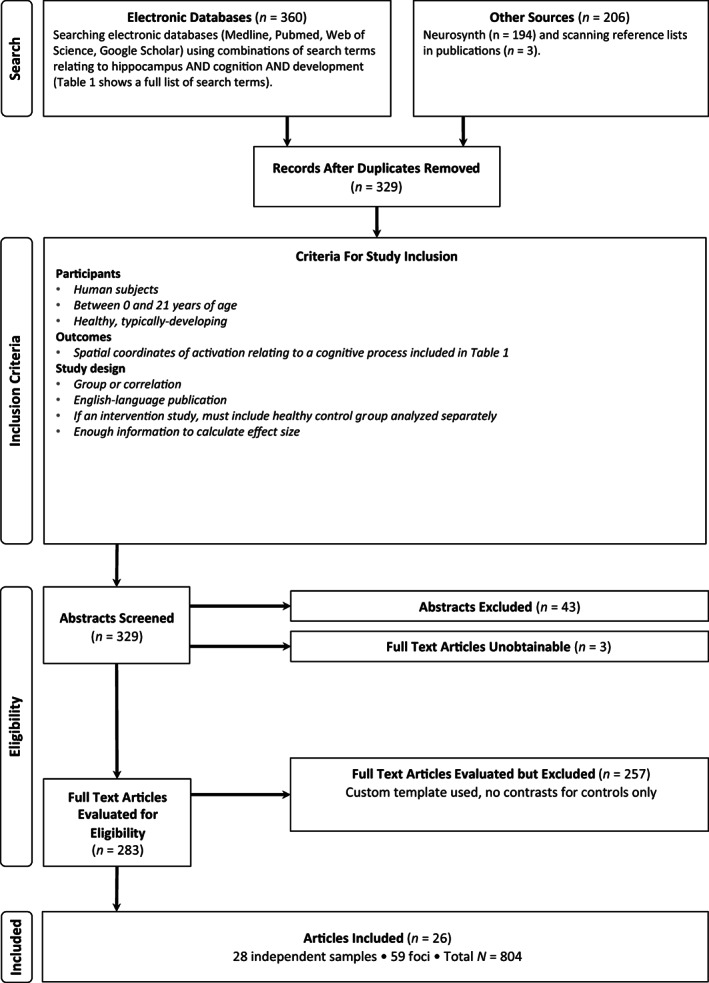
Flow diagram of search strategy.

This meta‐analysis was preregistered on Open Science Framework (https://osf.io/fwty5) prior to beginning. Literature searches were conducted using Medline, Pubmed, Neurosynth, and Web of Science without restriction on dates. We also searched Google Scholar, and scanned reference lists of relevant publications. The search strategy is shown in Table 1 and Figure 1. The list of cognitive processes included in our search strategy was developed by amalgamating broader keywords used to describe memory and emotion. A second search was completed in Neurosynth, which provides sets of “topics” created by applying Latent Dirichlet allocation (LDA) to the abstracts and text in the database (see Poldrack et al., 2012, for full methods). Briefly, Poldrack and colleagues applied LDA to a large set of neuroimaging publications, estimating the latent topics by creating groups of words that appear frequently together. Using a cross‐validated approach, they found that 130 topics best describes the neuroimaging literature. We completed this search using the full list of keywords included in topics 020, 048, 102, 229, 254, 256, 272, and 348. This list includes 320 keywords (with overlap), and the full set is included in supplementary materials Appendix 1. We classified studies into an “Emotion” category and a “Memory” category. The emotion category included the last four keywords listed under Cognitive process in Table 1, namely, faces, emotion, affect, motivation, as well as Poldrack topics 020, 048, 254, and 348. The memory category included all other words listed under Cognitive process in Table 1, and Poldrack topics 102, 229, 256, and 272. Face studies were screened to ensure they involved emotional faces or an emotion‐related task.

**TABLE 1:** Literature search terms used in Medline, Pubmed, Web of Science, and Google Scholar searches. To be indexed, studies needed to mention at least one term from each column (i.e., hippocampus AND cognitive process AND development).

Hippocampus	Cognitive process	Development
Hippocampus	Memory	Develop*
HPC	Spatial	Child*
	Semantic	Pediatric
	Episodic	Paediatric
	Pattern completion	Adolescen*
	Pattern separation	
	Encoding	
	Retrieval	
	Navigation	
	Gist	
	Detail	
	Recall	
	Faces	
	Emotion	
	Affect	
	Motivation	

* Represents a wildcard, where it can be substituted for any other number of characters.

A study was included for meta‐analysis only if it was performed in a healthy, typically developing human sample with participants ranging from 0 to 21 years of age, inclusive. Intervention studies were considered if there was a healthy control group whose data were analyzed separately to that of the intervention group. No data from intervention or patient groups were included in the present meta‐analysis. For a study to be included, it had to include the spatial coordinates of activation within the hippocampus for the cognitive tasks listed in Table 1.

All task‐based fMRI studies that met the above criteria were considered, regardless of task variation or stimuli type. Coordinates were analyzed in MNI space; if Talairach space is used, coordinates were transformed. No lower limit was placed on the timeframe of studies considered. Studies were considered from any article published in an English‐language, peer‐reviewed academic journal up to the present date. Unpublished reports were included in the form of theses or conference proceedings. If the necessary data were not directly available in the article, thesis or conference proceeding, corresponding authors were contacted by email. Resting‐state fMRI, magnetoencephalography, and positron emission tomography studies were excluded.

Records were managed using an Excel spreadsheet, specifically using Templates 7 and 8 provided by Moreau and Gamble (2020) for the literature search and the data extracted. Two independent reviewers (EK, JS) were engaged in the search of the study records. Each researcher conducted an independent literature search and decided on eligibility, and results were compiled into a single excel spreadsheet. The decision to include a study was made if both independent reviewers agreed on its eligibility based on the criteria listed above. Data from included studies were manually extracted from tables available in published manuscripts, and compiled in a table managed in Google sheets. Data for the following variables were extracted: mean and SD of age range of sample, sex ratio, cognitive process being studied, modality, and task. Data for the outcome variable of spatial location in coordinates of activation within the hippocampus were extracted.

### 2.2 Data synthesis

Data were analyzed using the ALE coordinate‐based meta‐analytic method (Eickhoff et al., 2009; Laird et al., 2005; Turkeltaub et al., 2002), available through BrainMap (http://brainmap.org/ale/; Research Imaging Center of the University of Texas in San Antonio). Contrast coordinates (i.e., foci) from different studies were used to generate 3D maps describing the likelihood of activation within a given voxel in a template MRI (Laird et al., 2005). Significant findings were based on whether the data are more likely to occur compared to a random spatial distribution.

Coordinates from source datasets were first transformed into common space. MNI coordinates were transformed into Talairach space using the best‐fit MNI‐to‐Talairach transformation (Lancaster et al., 2007). Random‐effects analyses were performed using GingerALE v3.0.2 (Eickhoff et al., 2009). Using this method, activation foci from each study were converted into three‐dimensional Gaussian probability functions. This process involved smoothing the data using a Gaussian blurring kernel. The full‐width at half maximum size of the Gaussian blurring kernel was based on the number of participants used in each contrast. A voxel‐wise likelihood of activation was calculated and corrected for multiple comparisons using the false discovery rate (FDR) *q* = 0.01.

### 2.3 Regional and laterality indices

**FIGURE 2:**
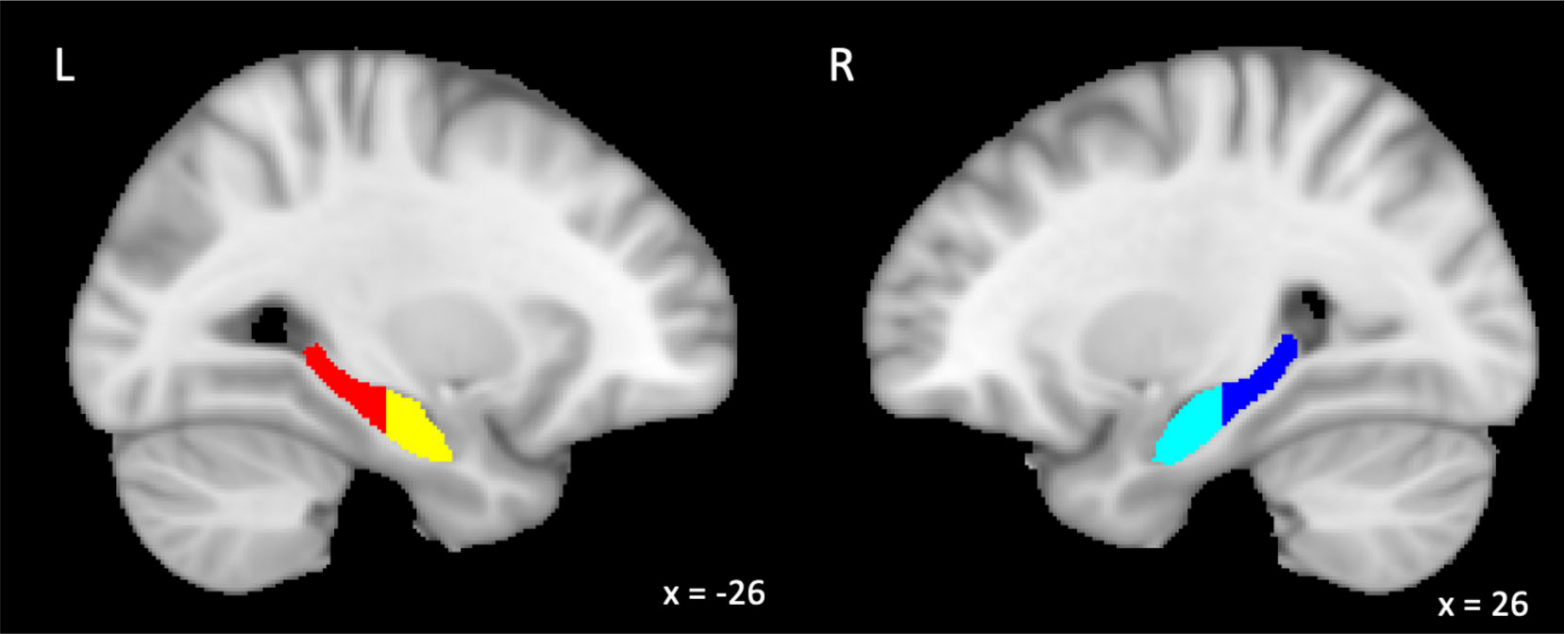
Segmentation of the anterior and posterior divisions of the left (yellow/red) and right (light/dark blue) hippocampus in MNI space.

The anterior and posterior regions of the hippocampus were drawn on the hippocampal segmentation from the Harvard‐Oxford subcortical structural atlas (Desikan et al., 2006; Frazier et al., 2005; Goldstein et al., 2007; Makris et al., 2006) in MNI template space using the protocol most commonly used in hippocampal head/tail segmentation (Malykhin et al., 2007; Poppenk et al., 2013; Yushkevich et al., 2010), and is shown in Figure 2. The border between the anterior and pHPC was defined by the presence or absence of the uncus. Specifically, the last slice in which the uncus is visible was defined as the last slice of the aHPC. On the MNI template, at or anterior to *y* = −21 was considered aHPC (Poppenk et al., 2013).

Hemispheric dominance of memory and affective processes was assessed by calculating laterality indices for each of the thresholded ALE maps. The thresholded ALE values within each hemisphere of the hippocampus regions‐of‐interest (ROIs) were summed. A laterality index (LI) was calculated [Right – Left]/[Right + Left] based on the summed values (Seghier, 2008). Hemispheric dominance was set at −0.2/0.2 (left/right) based on the thresholding methods used in previous literature (Duerden et al., 2013). A value less than −0.2 was deemed left hemisphere dominant, and a value greater than 0.2 was deemed right hemisphere dominant. Values in between were considered bilateral.

## 3 RESULTS

### 3.1 Demographic information

The 13 memory studies included 455 participants (45.3% female), the mean of the mean age of the participants was 12.2, and included ages ranged from 6 to 21. Two of the studies included only boys. Handedness was reported in 12 of the studies, and participants were 98.8% right‐handed. The 13 emotion studies included 26 separate contrasts and 349 participants, and sex ratio was reported for the contrast of interest in 10 studies, with 57.9% of participants being female. Mean age was reported for the contrast of interest in 11 studies, with the mean of the mean age of participants being 12.4. Included ages ranged from 4 to 21. Handedness was reported in 8 of the studies, and participants were 95.7% right‐handed. Mean age did not significantly differ between memory and emotion studies (*t*(19) = 0.21, *p* = .832).

### 3.2 Memory

**FIGURE 3:**
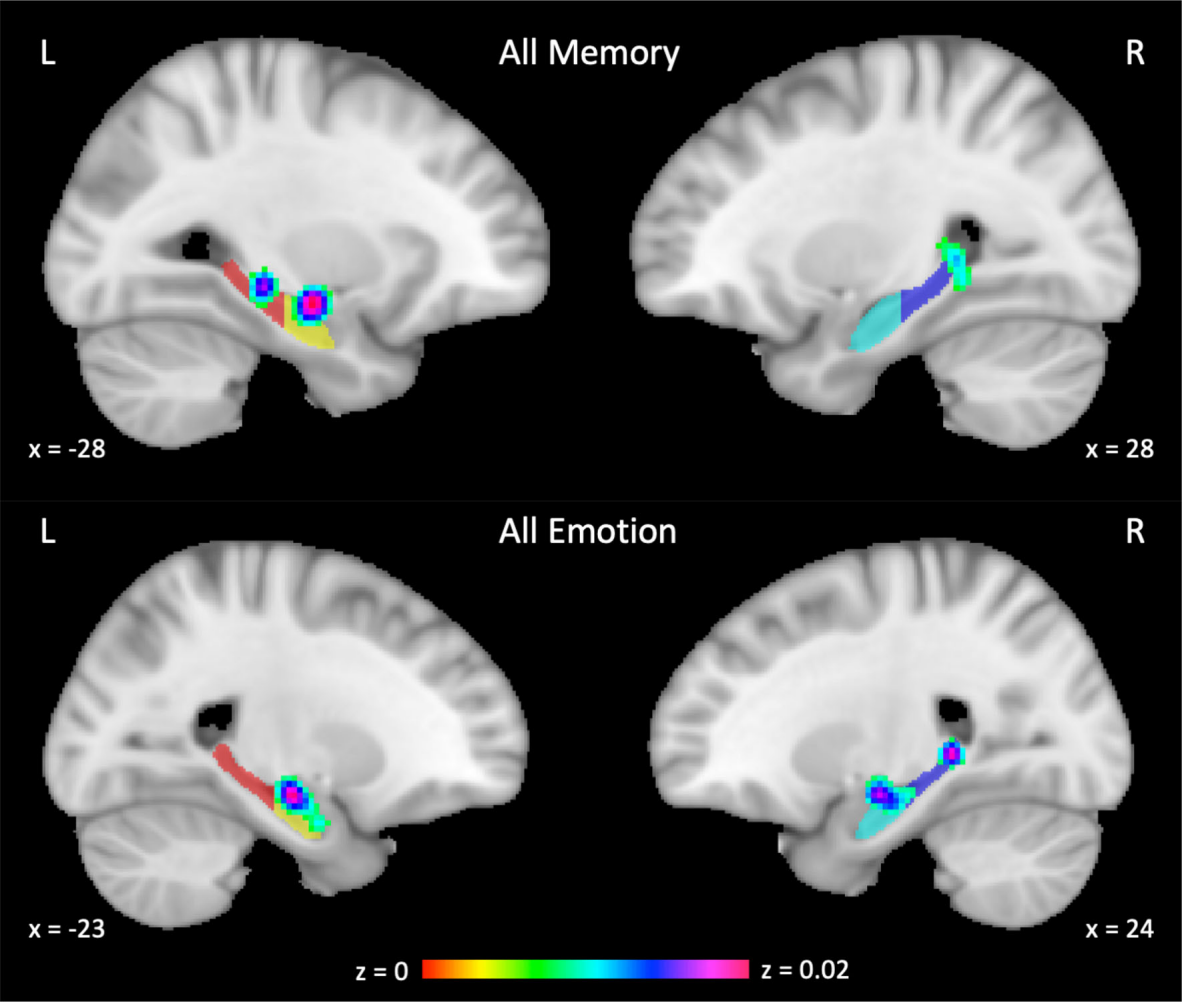
Activation likelihood estimate (ALE) maps for all memory contrasts and all emotion contrasts. Coordinates are in MNI space and overlaid on the anterior/posterior segmentations, L = Left. Clusters were thresholded at a family‐wise error rate of 0.05.

**FIGURE 4:**
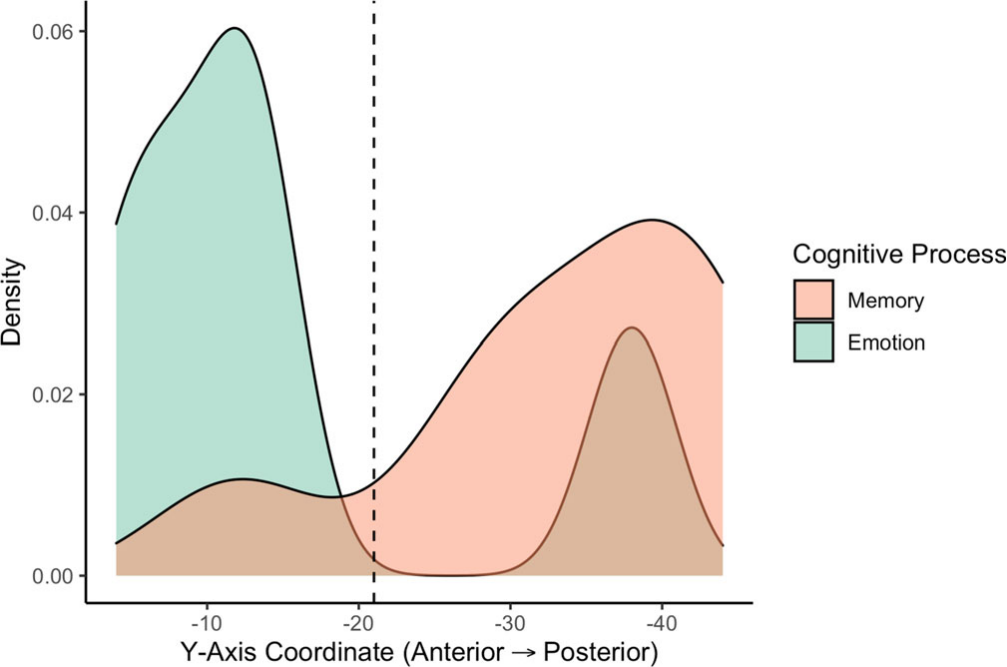
Distribution of peak cluster coordinates in the hippocampus along the *y*‐axis for memory and emotion. The *y*‐coordinate between anterior and posterior portions of the hippocampus is denoted by the dashed line.

**FIGURE 5:**
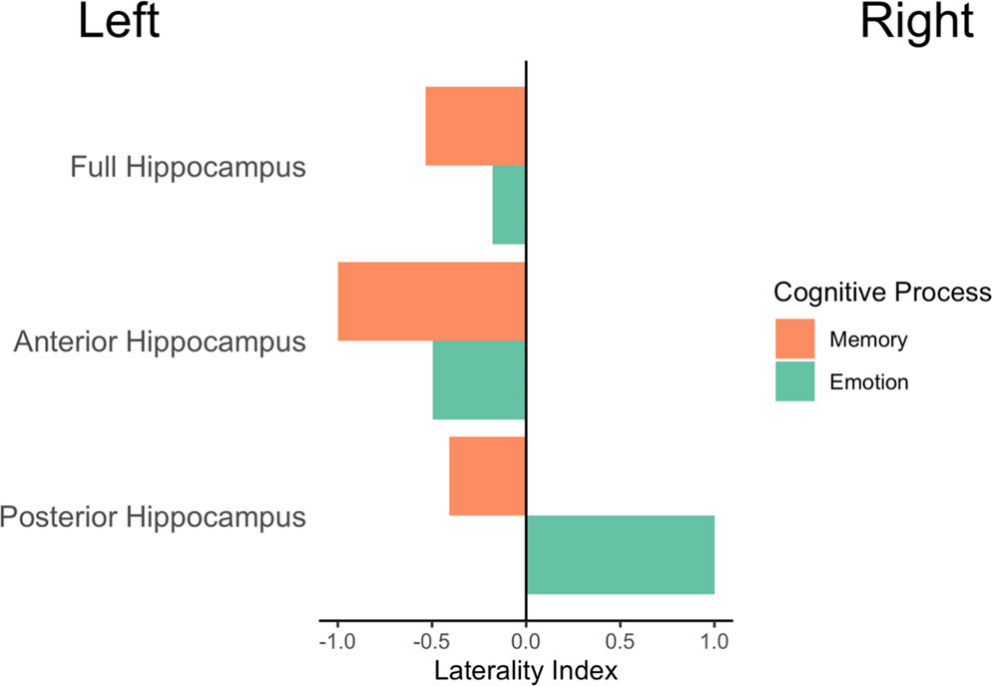
Laterality indices for memory and emotion within the full, anterior, and posterior hippocampus.

Studies included in the memory meta‐analysis are shown in Table 2. A total of 20 foci were extracted from the 13 contrasts reporting activation in the hippocampus in response to a memory task in 13 studies. The nature of the memory tasks was mostly encoding and retrieval presented visually, including autobiographical, declarative, and spatial memory. Most contrasts were retrieval‐ and recall‐based, including retrieval versus control, remembered versus forgotten targets, and hits versus misses. Then, 7 of the 13 contrasts included a form of verbal task (Bartha‐Doering et al., 2018; Bauer et al., 2017; Buck et al., 2020; Carrión et al., 2010; Herting & Nagel, 2013; Maril et al., 2010; Takashima et al., 2019). Three significant clusters of activation within the hippocampus in response to all memory tasks were found (Table 3, Figure 3). One cluster was in the left aHPC, whereas the remaining two clusters were found in the bilateral pHPC. Anterior/posterior localization of the significant clusters is shown in Figure 4, which plots the *y*‐coordinate of all extrema contained within each significant cluster found in Table 3. Laterality indices indicated that within the hippocampus, the left hemisphere was more activated by all memory stimuli (LI = −.53), plotted in Figure 5 for the whole, anterior, and pHPC.

**TABLE 2:** List of studies included in memory meta‐analysis.

Study #	Author(s)	Year	*N*	F	Age (range)	Tesla	Task	Contrasts
1	Bartha‐Doering et al.	2018	30	12	10.3 (7–16)	3	Auditory description definition task	Task vs. baseline
2	Bauer et al.	2017	14	7	10.3 (8–11)	3	Autobiographical and semantic memory retrieval	AM vs. rest, early vs. late AM retrieval, semantic retrieval
3	Buck et al.	2020	28	17	14 (8–18)	3	Noun‐to‐verb generation and cued recall	Memory encoding, block design; memory retrieval, event design
4	Carrión et al.	2010	11	4	13.9 (11–16)	3	Verbal declarative memory	Retrieval vs. control
5	Chen et al.	2016	9	4	10 (10–10)	3	*n*‐Back	Pre vs. post exercise
6	Cho et al.	2012	121	54	8.2 (7–9.9)	3	Arithmetic problem solving	Retrieval fluency correlation
7	Güler and Thomas	2013	30	14	8.7 (8–9) and 12.7 (12–13)	3	Paired‐associates picture memory task	Successful recall vs. forgotten
8	Herting and Nagel	2013	17	0	16.2 (15–19)	3	Subsequent memory paradigm	Remembered vs. forgotten words
9	Li et al.	2014	27	0	10.9 (6–15)	3	Categorical *n*‐back	Correct response vs. baseline
10	Maril et al.	2010	24	15	13.8 (7–19)	1.5	Verbal memory encoding	Remembered vs. forgotten words
11	Ofen et al.	2012	69	34	14.7 (8–21)	3	Memory retrieval of scenes	hits vs. misses
12	Sneider et al.	2018	32	15	13.9 (13–14)	3	Virtual Morris water task	Retrieval/hidden vs. motor/visible
13	Takashima et al.	2019	43	30	9.8 (8–10) and 15.5 (14–16)	3	Lexical decision	Day 1 vs. Day 8, Japanese

**TABLE 3:** Meta‐analyses results for memory and emotion studies.

Condition	Left/right	Anterior/posterior	Cluster #	*x*	*y*	*z*	Volume (mm^3^)	ALE value	*p*‐Value
Memory	Left	Posterior	1	−26	−28	−8	2200	0.01591701	*p* < .0001
	Left	Anterior	2	−30	−12	−14	1608	0.02018569	*p* < .0001
	Right	Posterior	3	28	−40	2	648	0.01118116	*p* < .0001
Emotion	Left	Anterior	1	−26	−14	−14	3040	0.01958763	*p* < .0001
	Right	Anterior	2	24	−12	−14	1264	0.01297942	*p* < .0001
	Right	Posterior	3	24	−38	0	608	0.01061688	*p* < .0001

### 3.3 Emotion

Studies included in the emotion meta‐analysis are shown in Table 4. A total of 39 foci were extracted from the 26 contrasts reporting activation in the hippocampus in response to an emotion task in 13 studies. The nature of the emotion experiments included emotional face processing, induction of an emotion such as shame, guilt, or reward, and emotion regulation. Most contrasts examined emotional (e.g., angry, fearful, disgusted) versus neutral faces, with others comparing threatening versus neutral targets, risky versus sure scenarios, and correlations with feelings of guilt. Three significant clusters of activation within the hippocampus in response to all emotion tasks were found (Table 3, Figure 3). Two clusters were in the bilateral aHPC, whereas the remaining cluster was found in the right pHPC. Anterior/posterior localization of the significant clusters is shown in Figure 4, which plots the *y*‐coordinate of each significant clustebastinr found in Table 3. LIs indicated that within the hippocampus, activation by all emotion stimuli was bilateral (LI = −0.17, Figure 5).

**TABLE 4:** List of studies included in emotion meta‐analysis.

Study #	Author(s)	Year	*N*	F	Mean age (range)	Tesla	Task	Contrasts
1	Bastin et al.	2021	36	36	18.8 (15–21)	3	Shame and guilt induction paradigm	Correlation with guilt ratings during negative feedback
2	Christensen et al.	2015	7	–	– (7–12)	3	Dot‐probe attention‐orienting task	Angry vs. blank
3	Doyle‐Thomas et al.	2013	16	0	14.69 (13–18)	3	Emotional matching	Visual > audiovisual
4	Karim and Perlman	2017	30	–	7.6 (4–12)	3	Emotional video viewing	Negative vs. positive, positive vs. negative
5	May et al.	2004	12	7	13.25 (8–18)	1.5	Reward‐based guessing game	Task vs. baseline
6	Miller et al.	2020	21	14	10.43 (8–12)	3	Social feedback processing task	Angry > smiling
7	Moore et al.	2012	45	26	13.1 (13–13)	3	Emotional face observation	Correlation with pubertal development during neutral face viewing
8	Paulsen et al.	2012	17	8	14.8 (14–16)	3	Risky decision‐making task	Risky > sure
9	Pfeifer et al.	2008	16	7	10.2 (9–10)	3	Emotional face observation	Observation of emotional vs. neutral expressions
10	Reidy et al.	2016	15	0	8.67 (7–11)	3	Emotional face observation	Disgust > neutral
11	Romund et al.	2016	83	43	14.48 (13–16)	3	Emotional face‐matching paradigm	Fearful vs. neutral
12	Steele et al.	2018	24	24	– (13–16)	3	Implicit emotion regulation task	Threatening vs. neutral targets
13	van Meer et al.	2016	27	–	10.9 (10–12)	3	Food‐viewing	Unhealthy vs. healthy

## 4 DISCUSSION

In the present meta‐analysis, we sought first to localize emotional and non‐emotional memory processing within the hippocampus in typically developing children, and second to determine whether there was a long‐axis hippocampal divide similar to adults, with emotion being localized to the aHPC. Results indicated that overall emotion was localized to the aHPC, with memory being localized to the pHPC. Additionally, we examined the laterality of both processes, and found that while memory was left dominant, emotion was processed bilaterally.

The finding that the aHPC is uniquely involved in emotion has long been emphasized in the nonhuman animal literature (Fanselow & Dong, 2010; Vogel et al., 2020), and was recently replicated in a meta‐analysis of adult human neuroimaging studies (Grady, 2020). However, while some studies suggest long‐axis differentiation in aspects of cognition may occur early in development (e.g., episodic memory versus statistical learning), differentiation for emotional processing has not been explored until now. Our results show that this broad functional specialization is also present in development. In addition to long‐axis functional organization, we also see functional differences in lateralization between emotion and memory. In what follows, we summarize evidence of long‐axis specialization in adults in the domains of emotion and memory, and compare it to our findings in children. We then turn to what is known about lateralization of emotion processing, and lateralization of hippocampal function, highlighting the novelty of our results and need for further work in this area.

### 4.1 Hippocampal functional organization of emotion

In rodents, lesions of the ventral (homologue of aHPC) but not dorsal (homologue of pHPC) lesions lead to emotional and stress responses (Henke, 1990). For example, ventral hippocampal modulations can decrease fear and anxiety (Kjelstrup et al., 2002; Maren & Holt, 2004), and increase motivation for food (Ferbinteanu & McDonald, 2001). In meta‐analysis of adult neuroimaging studies, social and emotional processing was localized to the aHPC (Grady, 2020). These patterns are supported by the differential connectivity of the aHPC. Animal tracing studies have revealed dense connections are present between the amygdala and the aHPC (but not pHPC) (Canteras & Swanson, 1992; Fanselow & Dong, 2010; Groenwegen et al., 1987; Henke, 1990; Strange et al., 2014; van Groen & Wyss, 1990). The aHPC is also more connected to the temporal, orbitofrontal, and anterior cingulate cortex, that is, areas associated with motivational behavior (Adnan et al., 2016; Vogel et al., 2020). Similar differential connectivity patterns have been found in humans between aHPC and the amygdala, orbitofrontal cortex, ventromedial prefrontal cortex, caudate, perirhinal cortex, and temporal pole (Grady, 2020; Kahn et al., 2008; Qin et al., 2016; Wang et al., 2016). Indeed, it has been proposed that the aHPC is part of an anterior‐medial network, whose primary function is processing of unique entities, including their affective and social importance (Ranganath & Ritchey, 2012). Currently, it is unknown when this system emerges developmentally and becomes adult‐like. While our results do not speak to functional connectivity of the system, they do show that functional specialization thought to result from this connectivity is present in development. If driven by underlying network connectivity, this provides evidence that the anterior‐medial system is present in development, supporting previous work (Blankenship et al., 2017; Riggins et al., 2016), and adds to existing theories of early functional specialization in the long‐axis, which have focused on different rates of intrinsic maturation between the anterior and posterior regions.

### 4.2 Hippocampal functional organization of memory

Due to the relatively small amount of developmental neuroimaging studies including the hippocampus, we collapsed a variety of nonemotional memory tasks into a general “memory” category, guided by topics by Poldrack et al. (2012). Activation in the memory category was largely localized within the pHPC. In general, our findings in this developmental sample (i.e., in ages 4–21) replicate meta‐analytic findings from adults (Grady, 2020) and animals (Fanselow & Dong, 2010).

Rodent work has shown that lesions to the dorsal hippocampus selectively impair encoding and retrieval of spatial memories (Moser et al., 1995). Additionally, evidence from human studies converge on the importance of the pHPC for spatial memory and navigation, but extend its functional specialization to other domains, such as scene processing, detailed recollection, episodic simulation, and more general retrieval (see Poppenk et al., 2013 for a review). This functional specialization is thought to be largely due to distinct connectivity patterns in the pHPC. Tracing studies in rodents have shown dorsal hippocampus is differentially connected to parahippocampal cortex, retrosplenial areas, and anterior cingulate cortex (Burwell & Amaral, 1998a, 1998b).

In humans, pHPC is functionally connected to parahippocampal cortex (Kahn et al., 2008; Libby et al., 2012), cuneus, precuneus, anterior and posterior cingulate cortex, and parts of the thalamus (Poppenk & Moscovitch, 2011). Similarly, Grady (2020) found stronger connectivity between pHPC, medial occipital cortex, posterior parietal cortex, thalamus, and lateral inferior frontal gyrus. Ranganath and Ritchey (2012) suggest that this distinct connectivity places the pHPC in a posterior‐medial network, specialized for the “situation models” or detailed representations of events, and that this shapes its involvement in a variety of memory and cognitive tasks. This is further supported by meta‐analytic findings that pHPC is most distinctly activated for recollection, spatial tasks, episodic simulation, navigation, and spatial processing, all of which rely on detailed spatio‐temporal representations.

Our memory category included some tasks in areas that align closely with these areas (spatial, episodic, pattern separation, retrieval, navigation, detail, recall) and others less clearly associated with pHPC (semantic, gist, pattern completion, encoding), yet we still found largely posterior localization. Thus, while our results generally show these same distinctions are present in development, there is currently not enough data to examine more subtle variations. For example, whether the memory tasks including search words such as semantic, pattern completion, and gist are actually associated with aHPC in development requires more data to investigate.

### 4.3 Lateralization

Existing data on lateralization of function in the hippocampus paints a less clear picture than what is known about long‐axis organization. At the scale of the entire brain, emotion has long been conceptualized as lateralized. For example, the right‐dominant hypothesis holds that all emotional processing is right‐hemisphere dominant, and was supported initially by the clinical observation that patients with left‐lateralized lesions retained emotion‐related language despite severe general language deficits (Jackson, 1878). Alternatively, the valence‐lateralization hypothesis argues that the right hemisphere is involved specifically in negative valenced emotions, while the left‐hemisphere is involved in positively valenced emotions (see Palomero‐Gallagher & Amunts, 2022 for a review). Recent data‐driven meta‐analytic approaches have revealed that the perception, experience, and expression of emotion are each subserved by a distinct large‐scale network (Morawetz et al., 2020), and that these networks are composed of regions which are left‐lateralized, right lateralized, and bilateral. In line with this, a meta‐analysis of 65 neuroimaging studies found no clear overall evidence for lateralization for emotion, and concluded lateralization is complex, region specific, and dynamic (Wager et al., 2003).

There is evidence of lateralization of verbal memory to the left hemisphere, and spatial processing to the right hemisphere (with complications and caveats). Left hemisphere stimulation in epileptic patients impairs word but not face recognition (Coleshill et al., 2004), and verbal memory is more impaired in individuals with left hippocampal damage than right (Saling et al., 1993). This language‐related lateralization is not simply material specific; individuals with left temporal lobe epilepsy are impaired in face processing when the task requires naming, even though they perform similarly to controls in face recognition, while individuals with right lateralized epilepsy were impaired in all face processing (Seidenberg et al., 2002). Evidence from a meta‐analysis examining cognitive outcomes following temporal lobe resection revealed greater deficits in verbal memory were associated with left‐sided surgery compared to right (Sherman et al., 2011). Later fMRI studies have replicated these results, indicating that the left hippocampus plays an important role in verbal memory (Jansen et al., 2009; Sidhu et al., 2015). In the domain of spatial cognition, some evidence suggests the right hippocampus is specialized (Burgess et al., 2002; Maguire et al., 1997, 1998). However, more recent works suggests both left and right hippocampus are involved in different aspects of spatial cognition, with the left hippocampus more involved in contextual memory (object locations) relative to navigation and tests of topographical memory (map drawing, scene recognition) which depended more on the right hippocampus (J. Miller et al., 2018; Spiers et al., 2001). In terms of hippocampal laterality of function in children, there is only one study which found greater right hippocampal involvement in children during a relational memory task (Hopf et al., 2013).

We found that our broad category of memory tasks activated the left hemisphere more than the right, in both aHPC and pHPC. This left‐lateralization of memory may result from the verbal nature of several of the tasks; 7 of the 13 studies involved some form of verbal, lexical, or language‐related stimuli. For example, two of the studies examined remembered versus forgotten words, one involved auditory descriptions, and another was a lexical decision for recently learned words. In contrast, the emotion laterality analysis showed bilateral hippocampal involvement in emotion processing.

Overall, these findings suggest that memory processing may depend more on the left hippocampus than the right, potentially due to the verbal material or task demands in most studies. This generally aligns with lateralization of verbal materials and tasks reported in adults, and suggests this lateralization is present in development. Much less is known about lateralization of emotion processing in the hippocampus, but our finding that emotional processing is bilateral fits with recent findings that many regions which are part of emotion networks are activated bilaterally (Morawetz et al., 2020). Much future work will be needed to elucidate whether specific components of cognition, or types of tasks involving emotion are lateralized, whether this occurs across the long‐axis or within anterior and posterior regions, and whether this differs across development.

### 4.4 Task‐related localization

When examining the laterality and anterior/posterior localization of the hippocampal coordinates of each memory study, no clear pattern emerged. For example, memory encoding activated left anterior (Maril et al., 2010), left posterior (Buck et al., 2020), and right posterior (Herting & Nagel, 2013) hippocampus. When examining memory retrieval, ROIs included left anterior (Bauer et al., 2017; Buck et al., 2020), left posterior (Bauer et al., 2017; Sneider et al., 2018), and right posterior (Carrión et al., 2010; Cho et al., 2012) hippocampus. Tasks that relied on verbal memory reported coordinates in the left anterior (Buck et al., 2020; Maril et al., 2010), left posterior (Bauer et al., 2017; Buck et al., 2020), and right posterior (Bartha‐Doering et al., 2018; Bauer et al., 2017; Carrión et al., 2010; Herting & Nagel, 2013; Takashima et al., 2019) hippocampus. Similarly, no clear pattern emerged for the localization of different types of emotion tasks. For example, emotional face viewing activated the left anterior (Christensen et al., 2015; Miller et al., 2020; Moore et al., 2012), left posterior (Romund et al., 2016), and right anterior (Reidy et al., 2016) hippocampus. Reward‐based studies activated the left anterior (van Meer et al., 2016) and right posterior (May et al., 2004) hippocampus. The variability of the individual results highlights the importance of meta‐analyses, which allow us to pool results and see whether patterns emerge. In the future, larger meta‐analyses should be conducted so that finer‐grained investigations can be conducted into whether in general, certain types of tasks within the memory and emotion domains can be localized.

### 4.5 Deviations from preregistration

Although the preregistration plan was followed closely, there were some realities associated with the data that meant that certain steps could not be performed. First, Objective 3 was to investigate the emergence of functional specialization. As there were limited studies that met our criteria, and these studies covered large age ranges, we were not able to examine how specialization changed across development. Second, *p*‐curve adjustment could not be applied; localization is based purely on coordinates and sample size, rather than *p*‐values. However, FDR thresholding is performed on the statistical brain map created. In this method, the corrected significance of a particular voxel depends on the overall shape of the curve of the p‐values in the map, thus taking into account all *p*‐values in the statistical map (but not the original studies) when creating the final thresholded results.

## 5 CONCLUSIONS, LIMITATIONS, AND FUTURE STEPS

In sum, our finding that emotion activates the aHPC and more general memory the pHPC in children and adolescents reflects what has been reported in both animal and human adult work (e.g., Fanselow & Dong, 2010; Grady, 2020). However, the small number of studies conducted in children and adolescents that met our meta‐analytic criteria meant that we could not examine more fine‐grained distinctions between memory and emotion. Both memory and emotion are broad terms to describe numerous processes; for example, encoding, retrieval, subsequent memory, and spatial memory are all distinct processes that fall under the umbrella of *memory*, and emotion is equally multifaceted. Similarly, the small number of studies and the wide range of ages used in each one meant it was not possible to conduct more in‐depth analyses of how localization of activation varies with age. For example, studies by Buck et al. (2020) and May et al. (2004) both included participants aged 8–18, while Ofen et al. (2012), included participants aged 8–21, and Maril et al. (2010), included participants aged 7–19.

Although *thousands* of memory and emotion studies have been conducted in developmental samples, those that reported usable hippocampal coordinates in typically developing children and adolescents were relatively few. This is in part due to many studies examining patient populations in relation to healthy controls, as well as due to custom child‐based templates being used that could not be translated into standard coordinates. Additionally, child imaging is notoriously difficult due to the data loss caused by motion in the scanner, perhaps further decreasing the number of studies conducted. Nevertheless, despite small numbers of studies, final sample sizes for each meta‐analysis were large, and the results which demonstrate long‐axis specialization with regard to memory and emotion in children and adolescents similar to that seen in adults is an important contribution to the endeavor to fully model hippocampal organization. While our sample ranged in age from 4 to 21 years, future research should examine long‐axis specialization as a function of age to determine at what point in development this specialization emerges, or whether it is present from birth.

## CONFLICT OF INTEREST

The authors declare no conflicts of interest.

## Supporting information


**APPENDIX S1:** Supporting Information

## Data Availability

Data compiled from the studies, as well as the output of the meta‐analysis, are available on OSF: https://osf.io/v8m62/files/osfstorage/630503537eb6d315671fb3f5.
