# Supplementary material for: Hippocampus long‐axis specialization throughout development: A meta‐analysis
Source: Hum Brain Mapp. 2023 May 20;44(11):4211–24. doi: 10.1002/hbm.26340 (PMC10318218; doi:10.1002/hbm.26340)
Supplement: Supplementary file 1 — APPENDIX S1: Supporting Information [file HBM-44-4211-s001.docx]

Appendix 1

| Topic # | Terms |
| --- | --- |
| 020 | emotional, emotion, amygdala, neutral, negative, affective, positive, valence, emotionally, emotions, response, pictures, reactivity, regulation, impact, cognitive, influence, arousal, reactions, implicated, interaction, hippocampus, context, limbic, correlates, scanned, viewed, emotionality, prefrontal, appraisal, female, cues, presented, relevance, salience, psychiatric, valenced, cognition, evoking, modulated |
| 048 | faces, amygdala, emotional, fearful, neutral, happy, sad, facial, expressions, angry, face, response, emotion, fear, social, expression, threat, viewing, masked, perception, affective, presented, emotionally, gender, salient, bias, valence, limbic, versus, threatening, reactivity, affect, methods, responsiveness, viewed, presentation, anxiety, amygdalae, responsivity, anger |
| 102 | hippocampal, hippocampus, memory, ca, cc, formation, declarative, neocortical, episodic, parahippocampal, encoding, hippocampi, posterior, relationship, consolidation, functions, recall, learning, para, amnesic, amnesia, binding, mnemonic, perirhinal, preferential, volume, subiculum, jon, entorhinal, lesions, sbp, subfields, gradual, possibly, dg, disengagement, remnant, dentate, notably, lacking |
| 229 | mtl, recollection, memory, familiarity, recognition, temporal, medial, confidence, lobe, cortex, perirhinal, hippocampus, parahippocampal, item, true, prc, items, source, retrieval, phc, strength, test, remember, paradigm, episodic, suggesting, entorhinal, correct, subregions, decisions, recollected, hsv, studied, gist, success, lobes, episode, recollective, roles, metamemory |
| 254 | empathy, social, empathic, insula, affective, emotional, feelings, prosocial, interpersonal, ai, feeling, individuals, pain, emotions, anterior, states, sharing, experience, concern, taking, observing, perspective, emotion, affect, experiencing, distress, personal, compassion, suffering, trait, mentalizing, understand, experiences, situations, vicarious, embarrassment, physical, share, empathize, observation |
| 256 | encoding, memory, recognition, subsequent, successful, hippocampus, encoded, items, formation, test, remembered, retrieval, episodic, item, hippocampal, binding, forgetting, remember, forgotten, recognized, suggest, subsequently, success, predicted, memories, incidental, correlates, medial, material, verbal, studied, paradigm, function, recall, parahippocampal, successfully, scanning, tested, unsuccessful, shallow |
| 272 | retrieval, memory, episodic, memories, encoding, hippocampus, recall, retrieved, reactivation, successful, correlates, recollection, hippocampal, precuneus, retrieve, autobiographical, parahippocampal, retrieving, ams, semantic, reinstatement, success, test, events, remembering, declarative, search, encoded, past, linked, learned, correct, versus, remembered, mnemonic, recalled, laboratory, reactivated, paired, rich |
| 348 | facial, emotion, expressions, emotional, emotions, recognition, expression, fear, neutral, anger, amygdala, disgust, sadness, faces, affect, happiness, perception, face, affective, social, expressing, joy, static, viewed, happy, presented, insula, communication, gender, disgusted, intensities, fearful, recognize, sad, expressive, substrate, angry, images, fusiform, displaying |
